# Isolation of porcine adult cardiomyocytes: Comparison between Langendorff perfusion and tissue slicing-assisted enzyme digestion

**DOI:** 10.1371/journal.pone.0285169

**Published:** 2023-05-26

**Authors:** Xun Shi, Xiaoli Tang, Fang Yao, Le Wang, Mingzhi Zhang, Xin Wang, Guangxin Yue, Li Wang, Shengshou Hu, Bingying Zhou

**Affiliations:** 1 State Key Laboratory of Cardiovascular Disease, Fuwai Hospital, National Center for Cardiovascular Diseases, Chinese Academy of Medical Sciences and Peking Union Medical College, Beijing, China; 2 Shenzhen Key Laboratory of Cardiovascular Disease, Fuwai Hospital Chinese Academy of Medical Science, Shenzhen, Shenzhen, China; 3 Division of Prevention and Community Health, National Center for Cardiovascular Disease, National Clinical Research Center of Cardiovascular Disease, State Key Laboratory of Cardiovascular Disease, Fuwai Hospital, Peking Union Medical College, Chinese Academy of Medical Sciences, Beijing, China; 4 State Key Laboratory of Cardiovascular Disease, Center for Cardiovascular Experimental Study and Evaluation, National Center for Cardiovascular Diseases, Beijing Key Laboratory of Pre-clinical Research and Evaluation for Cardiovascular Implant Materials, Animal Experimental Center, Fuwai Hospital, Chinese Academy of Medical Sciences and Peking Union Medical College; University of Minnesota, UNITED STATES

## Abstract

Tissue slicing-assisted digestion (TSAD) of adult cardiomyocytes has shown significant improvements over conventional chunk methods. However, it remains unclear how this method compares to Langendorff perfusion, the current standard of adult cardiomyocyte isolation. Using adult Bama minipigs, we performed cardiomyocyte isolation via these two distinct methods, and compared the resulting cellular quality, including viability, cellular structure, gene expression, and electrophysiological properties, of cardiomyocytes from 3 distinct anatomical regions, namely the left ventricle, right ventricle, and left atrial appendage. Our results revealed largely indistinguishable cell quality in all of the measured parameters. These findings suggest that that TSAD can be reliably used to isolate adult mammalian cardiomyocytes as a reliable alternative to perfusion in cardiomyocyte isolation from larger mammals, particularly when Langendorff perfusion is not feasible.

## 1. Introduction

Cardiovascular diseases remain the leading cause of death worldwide. According to statistics published by the AHA, approximately 19.05 million people died of cardiovascular diseases in 2020 [1]. A robust model for studying cellular mechanisms of disease pathophysiology lies at the heart of cardiac research. Recent advancements in disease modeling suggest human primary cardiomyocytes as an efficient model in predicting drug responses. However, adult human primary cardiomyocytes are fragile in terms of their susceptibility to hypoxia and other isolation stress. In particular, the isolation of adult human cardiomyocytes from myocardial specimens is technically highly challenging due to its incompatibility with arterial perfusion with enzymatic digestion solution, a technique commonly used in rodent models.

Previous methods for the isolation of human cardiomyocytes have relied on chunk digestion, in which the specimen is cut into tissue chunks prior to digestion using the enzymatic digestion solution. Our group previously modified and optimized the protocol by introducing tissue slicing of the specimen in ice-cold cardioplegic buffer [2], and obtained satisfactory cell viability. Here, we sought to systematically characterize whether cardiomyocytes isolated via our protocol of tissue-slicing assisted digestion (TSAD) yielded cellular quality similar to that of Langendorff perfusion.

To this end, we compared these two techniques using Bama minipigs, whose cardiac anatomy and electrophysiology are very similar to humans. Specifically, we assessed cell viability, structure, gene expression and function of isolated cells from different compartments of the porcine heart. Our findings revealed TSAD as a robust method for the isolation of adult cardiomyocytes from large mammals.

## 2. Materials and methods

### 2.1. Animals and surgery

Six male Bama minipigs (male; age 10–11 mo; weight 28.42 ± 1.71 kg) were purchased from Beijing Farm Animal Research Center (BFARC), Institute of Zoology, Chinese Academy of Sciences, and housed at the Animal Experiment Facility of Fuwai Hospital (12:12-hour light-dark cycle, 20–26°C, 40–70% relative humidity, fresh air system) for 5–7 days prior to experiments for adaptation. All animal experiments comply with the ARRIVE guidelines and according to the Guide for the Care and Use of Laboratory Animals published by the National Institutes of Health, USA. All experimental procedures were approved by the Institutional Animal Care and Use Committee (IACUC), Fuwai Hospital, Chinese Academy of Medical Sciences.

Prior to surgeries, minipigs were randomly divided into Langendorff group and slicing-assisted digestion group. Anesthesia was induced with a tiletamine hydrochloride and zolazepam hydrochloride (Zoletil) (1 mg/kg), and maintained with xylazine hydrochloride (1 mg/kg), as well as inhalation of 0.2–2% isoflurane. A tracheal tube (8 mm-diameter) was inserted, and connected to a ventilator (Savina, Dräger, Germany). The animals were heparinised with 90–100 mg heparin, and muscle was relaxed using 50–90 mg succinylcholine. During surgery, the ascending aorta was clamped and hearts were perfused with a cold proprietary cardioplegic solution, and then excised upon cardiac arrest for the isolation of cardiomyocytes.

### 2.2. Isolation of porcine cardiomyocytes

For Langendorff perfusion, explanted hearts were mounted on a Maquet HL 20 perfusion system, and perfused with our modified Tyrode’s solution [3] for 10–15 min to wash out remaining blood. For digestion, oxygenated Ca2+—free Tyrode’s solution containing 275 U/ml collagenase type II (Worthingon, LS004176) was maintained at 37°C, and perfused at a constant rate of 200 ml/min for 1 h. Once the heart became palpably flaccid and pale, it was removed from the perfusion device, and the entire left atrial appendage (LAA), and portions of the right (RV) and left ventricular (LV) free wall were excised, individually triturated with surgical scissors and pipetted repeatedly to disperse cells. Cell suspensions were filtered through a 200 μm-nylon mesh, and centrifuged (100 × *g*, 3 min, 4°C) to collect cardiomyocytes. Pelleted cardiomyocytes were resuspended in transport buffer (10% FBS in Ca2+—free Tyrode’s solution), and subjected to further analyses.

For TSAD, LAA, RV and LV specimens collected and digested according to our published method with slight modifications [3]. Specifically, we used the University of Wisconsin solution (Belzer, CHD120419) for tissue transport and slicing. Tissue was sliced to a thickness of 300 μm, perfused with Ca^2+^-free Tyrode’s solution (pH = 7.4) using an enzyme solution containing 275 U/ml. Following enzymatic digestion, cells were filtered through a 200 μm-nylon mesh, collected by gentle centrifugation (100 × *g*, 3 min, 4°C), and resuspended in the same transport buffer as in the Langendorff group.

### 2.3. Cell viability assessment

Cell viability staining was performed using the LIVE/DEAD^™^ Viability/Cytotoxicity Kit (ThermoFisher, L3224), following the manufacturer’s instructions. Stained cells were imaged using a microscope (Leica, DMI4000B), and quantified by manual counting of green (live) versus red (dead) cells in ImageJ (v1.8.0). Cell viability was as follows: Cell viability = number of live cells/ total cell number × 100%. Of note, round cells with bright green fluorescent were considered apoptotic, and thus excluded from live cell count. For the calculation of the percentage of rod-shaped cells, bright-field cell images were counted manually using Image J. The percentage of rod-shaped cells was calculated as the fraction of cells maintaining the rod shape within the total cell population. We compared these two methods to propidium iodide (PI) staining using human primary cardiomyocytes, and showed that PI staining resulted in overestimation of cell viability (S1A and S1B Fig, S2 Data). Therefore, we only used LIVE/DEAD staining and percentage of rod-shaped cells for evaluation of cell viability.

### 2.4. Immunofluorescence staining

Cardiomyocytes were plated into 15 mm-confocal dishes for immunofluorescence staining. After removing culture medium, cells were rinsed once with DPBS, fixed with 4% paraformaldehyde for 10 min, and then permeabilized in 0.1% Triton X-100 for 7 min. The cells were washed 3 times with ice-cold PBS and incubated with a blocking solution containing 1% BSA, 22.5 mg/ml glycine and 0.1% Tween 20 in PBS. After blocking, cells were incubated with primary antibodies in a humidified chamber at 4°C overnight. Primary antibodies included anti-ACTN2 (abcam, ab9465, 1:200) and anti-TNNT2 (abcam, ab45932, 1:200) antibodies. After decanting the primary antibody solutions, cells were washed 3 times with PBS, and then incubated with secondary goat anti-rabbit-Alexa Fluor 488 (Thermo, A32731, 1:1000) or goat anti-mouse-Alexa Fluor 594 (Thermo, A11032, 1:1000) antibodies for 2–4 h at room temperature in the dark. Finally, cells were washed 3 times with PBS and mounted with DAPI (Thermo, P36981). Samples were imaged on a confocal laser scanning microscope (Leica SP8, Germany) using a 40× water immersion objective, with identical image capture settings for all groups. Sarcomere lengths were quantified using the Leica Application Suite X software (LAS_X_Core_3.7.2).

### 2.5. Transmission electron microscopy

Cardiomyocytes were prefixed with 2.5% glutaraldehyde at 4°C overnight, and fixed with 1% OsO_4_ for 2 h. Fixed cells were dehydrated in an ethanol gradient (50%, 70%, 80%, 90%, 95%, and 100%) for 15 min at each step. After dehydration, samples were embedded in EPON-812. Ultrathin sections of 70–90 nm thickness were cut using an ultramicrotome (Leica EM UC7, Germany) and stained with uranyl and lead citrate for 15 min. Finally, image observation was performed using a transmission electron microscope (JEM-1230, JEOL, Japan). Mitochondrial size and area fraction were measured using Image J (v1.8.0).

### 2.6. RNA sample preparation and RNA-seq

Isolated porcine cardiomyocytes were pelleted and flash frozen at -80° C for storage. Total RNA was isolated as previously described [3]. cDNA libraries were constructed using the KAPA mRNA HyperPrep Kit (Illumina, KK8581) and sequenced on a Illumina NextSeq 500 sequencer.

### 2.7. RNA-seq analysis

Bioinformatic analysis of RNA-seq data was performed as previously described [3]. To compare the gene expression of between samples isolated by either Langendorff or TSAD, we firstly calculated the average logTPM (transcripts per kilobase of exon model per million mapped reads) of the samples in each group. Then, genes were ranked by decreasing average logTPM values, and the top 500 ones were selected for further analysis. Next, we used VennDiagram (v1.7.3) in R to plot the overlapping and differential genes between the two isolation in the LV, RV, and LAA, respectively. To annotate the resulting gene subsets, KEGG pathway and GO enrichment analyses were conducted by clusterProfiler (v3.2.14, PMID:22455463) in R, and enriched biological process (BP) are plotted using Prism GraphPad (v8.2.1.441).

### 2.8. Action potential recording

Whole-cell patch clamping was performed using a bath solution containing (mM) NaCl 140, KCl 3.5, CaCl_2_ 2, MgCl_2_ 1, HEPES 10, glucose 10, and NaH_2_PO_4_ 1.25, pH 7.4 with NaOH, and a pipette solution containing (mM) NaCl 5, CaCl_2_ 0.1, Mg-ATP 2, HEPES 10, ATP 5, K-Gluconate 140, MgCl_2_ 1, and EGTA 1, pH 7.2 with KOH. Resting membrane potential was adjusted to approximately -80 mV via the injection of a negative offset current before APs recording. APs were elicited by injection of 1.1–1.2 times the threshold current pulses for 10 ms at a frequency of 1 Hz. Offline data analysis and statistical evaluations were performed with Clampfit 10.6 (Axon).

## 3. Results

### 3.1. Cell viability and morphology

We have previously reported on a new set of tissue slicing-based methods to improve isolation of adult human primary cardiomyocytes (hPCMs). However, tissue sectioning using the microtome could introduce mechanical stress, thus compromising cellular quality. Here, we used Bama minipigs to test the performance of tissue slicing-assisted digestion (TSAD) in comparison with Langendorff perfusion-based cardiomyocyte isolation. Porcine hearts share a spectrum of similarities with human hearts, including anatomy and electrophysiology, and thus are frequently used as big animal cardiac models and donors for xenotransplantation [4–7].

**Fig 1:**
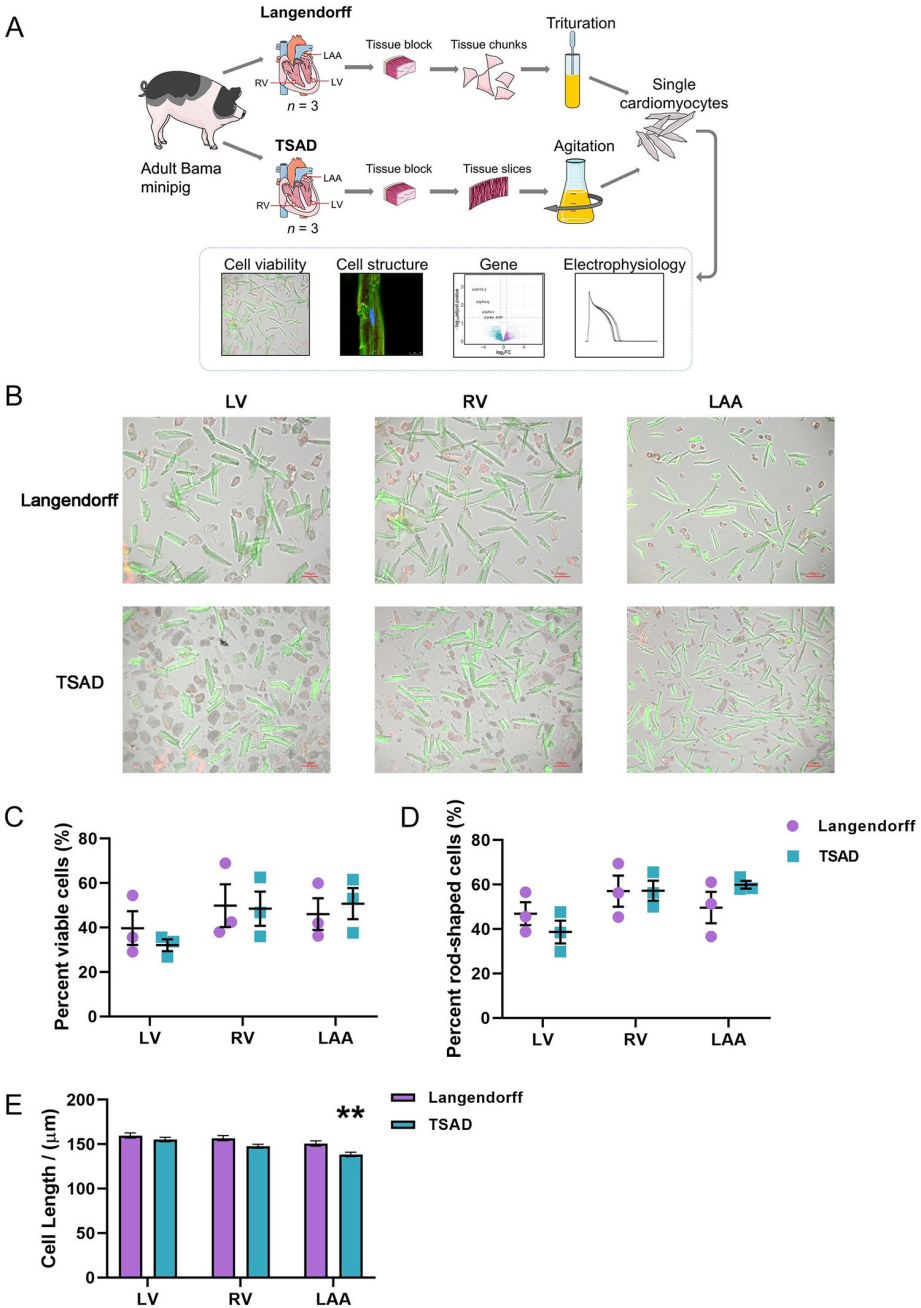
Cardiomyocyte viability and morphology. **(A)** Schematic of experimental design. Three pairs of pigs were subjected to either Langendorff perfusion or tissue slicing-assisted digestion (TSAD) for the isolation of cardiomyocytes from the left ventricle (LV), right ventricle (RV) and left atrial appendage (LAA). Isolated cells were subjected to a series of evaluations. **(B)** Representative images of cardiomyocytes stained using calcein-AM and EthD-1 following isolation. Scale bar = 100 μm. **(C)** Quantification of the percentage of viable cardiomyocytes in **(B)**. **(D)** Quantification of the percentage of rod-shaped cardiomyocytes from **(B)**. **(E)** Quantification of cell length in **(B)**. Data are mean ± SEM. ** *P* < 0.01, two-way ANOVA, compared to Langendorff.

The hearts of three pairs of pigs were removed from the chest, and either cannulated for Langendorff perfusion, or dissected for tissue slicing-assisted enzymatic digestion (TSAD). Cardiomyocytes (CMs) from three different anatomical regions, i.e., left ventricle (LV), right ventricle (RV), and left atrial appendage (LAA), were used for comparison (Fig 1A). The LV-, RV- and LAA-CM yields in the Langendorff group were 2.38×10^5^, 1.23×10^6^, and 2.47×10^6^ cells per gram of tissue, respectively, while those in the TSAD group were 1.33×10^5^, 1.33×10^6^, and 4.41×10^6^ cells per gram of tissue, respectively. Next, we assessed cell viability and cellular morphology. In general, the TSAD method gave rise to more tissue debris, as would have been expected for such a protocol involving slicing and cutting (Fig 1B). However, the viabilities of and the percentages of rod-shaped cells in TSAD-isolated CMs displayed no significant differences from the Langendorff group (Fig 1B–1D, S1 Data). Cell length measurements revealed that TSAD-isolated CMs showed decreasing trend in cell lengths compared Langendorff perfusion, but only reached statistical significance for LAA-CMs (150.7 ± 44.77 μm in Langendorff versus 138.6 ± 36.93 μm in TSAD, *P* = 0.0043) (Fig 1E). These data indicated that TSAD had little impact on cell viability and morphology of isolated CMs compared to Langendorff perfusion.

### 3.2. Cardiomyocyte structure and ultrastructure

**Fig 2:**
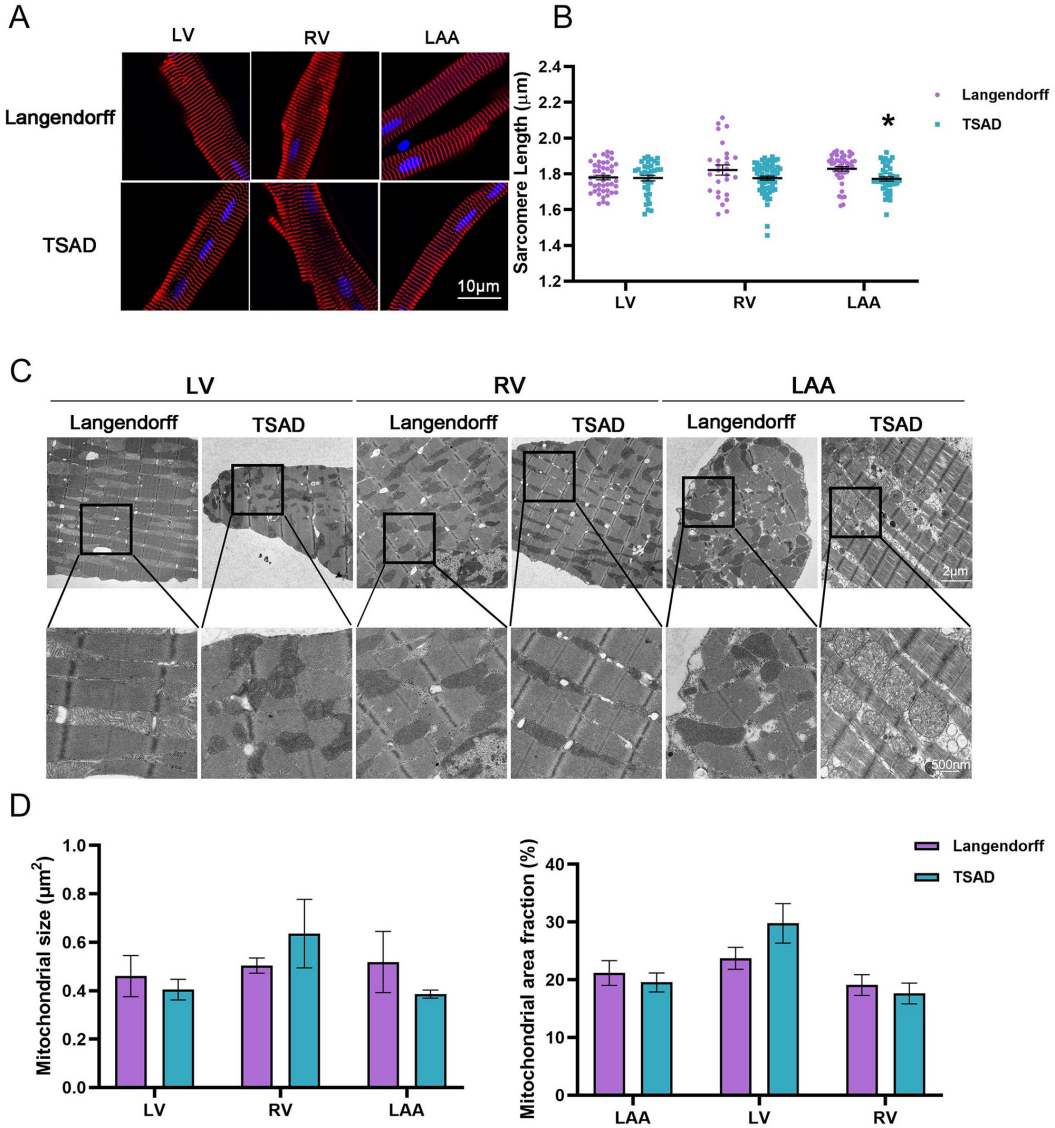
Cardiomyocyte structure and ultrastructure. **(A)** Immunofluorescence staining of ACTN2 (red) and DAPI (blue) in isolated porcine cardiomyocytes. Scale bar = 10 μm. **(B)** Quantification of sarcomere length in **(A)**. **(C)** Transmission electron microscopy (TEM) of porcine CMs. Scale bar = 2 μm (upper panel); = 500 nm (lower panel). Mitochondrial size and mitochondrial area fraction. **(D)** were quantified from 3 independent experiments. Data are mean ± SEM. * *P* < 0.05, two-way ANOVA, compared to Langendorff.

We further evaluated cellular structure by immunofluorescence staining of cardiac marker ACTN2 (α-actinin 2). CMs from both isolation groups displayed rod-shaped cell morphology with clear striations, suggesting comparable structural integrity (Fig 2A). Quantification of sarcomere lengths revealed similar lengths in LV and RV, but reduced lengths in TSAD-isolated LAA-CMs compared to Langendorff-isolated ones, which was consistent with cell length measurements (Fig 2B, S3 Data). Taking it a step further, we performed transmission electron microscopy (TEM) to determine potential ultrastructural differences between the two isolation methods. Overall, the majority mitochondria appeared unswollen and demonstrated healthy cristae structure. Langendorff-perfused LV-CMs generally displayed tightly packed arrays of myofibrils and well-organized sarcomeres and mitochondria, whereas TSAD-isolated LV-CMs exhibited a certain degree of mitochondrial derangement (Fig 2C). On the contrary, for LAA-CMs, Langendorff perfusion led to irregularly distributed mitochondria, whereas TSAD-isolated LAA-CMs showed normal ultrastructure. Similar observations were also made in the RV, where cardiomyocytes were structurally better preserved with TSAD (Fig 2C). To assess whether mitochondrial mass was affected by different isolation methods, we quantified mitochondrial sizes and the fraction of mitochondrial area within cells, but failed to identify significant differences in any of the regions (Fig 2D, S3 Data). Taken together, cardiomyocytes isolated via TSAD were generally structurally similar to those digested by Langendorff perfusion.

### 3.3. Cardiomyocyte gene expression

**Fig 3:**
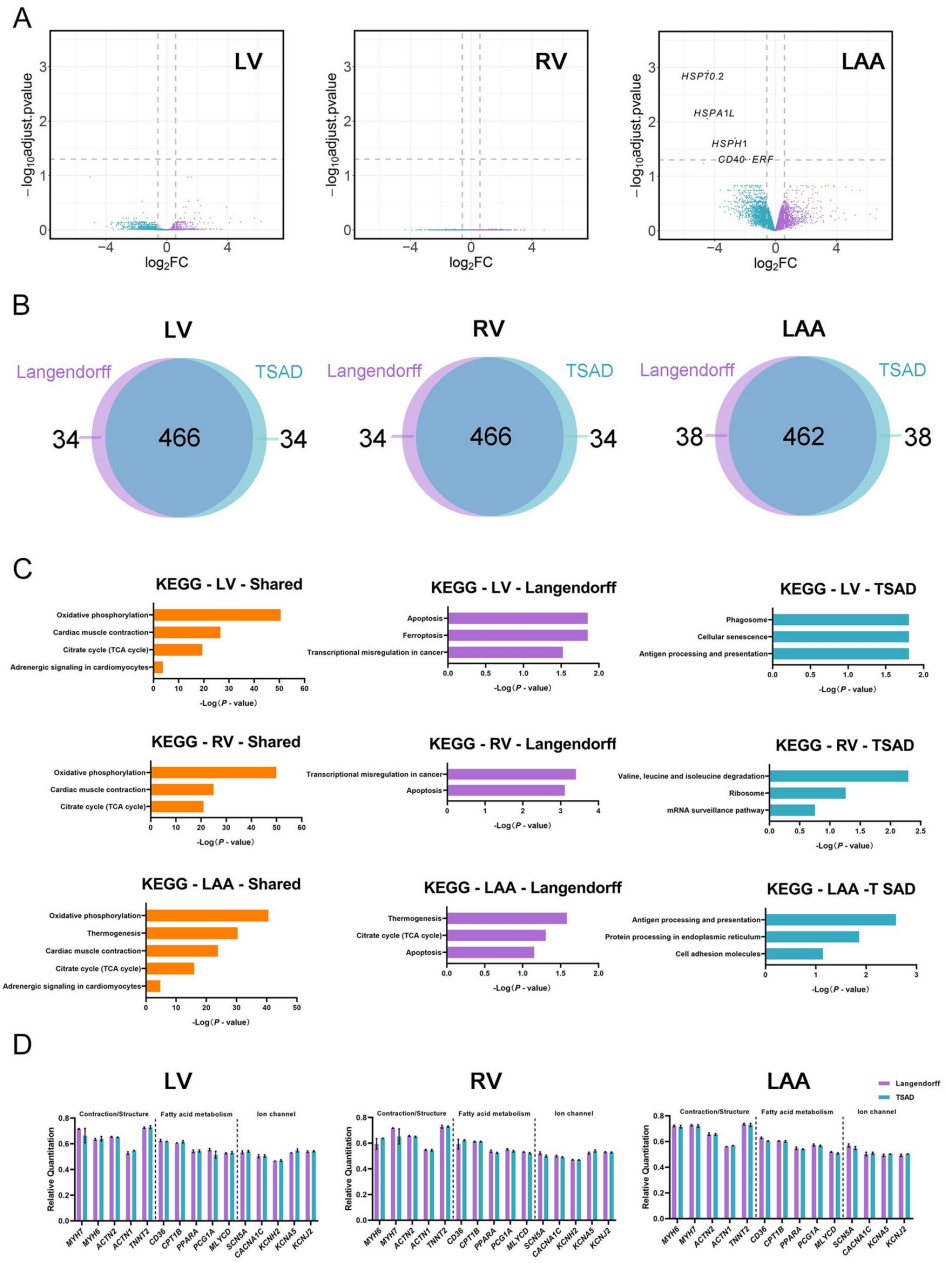
RNA-seq of isolated cardiomyocytes. **(A)** Volcano plots of DEGs between Langendorff and TSAD-isolated porcine CMs. Cyan, genes upregulated in TSAD group; violet, genes upregulated in Langendorff group. **(B)** Venn diagram showing the number of shared and distinct genes between Langendorff and TSAD groups within the top 500 expressed genes for LV, RV and LAA, respectively. **(C)** Functional enrichment (KEGG) of shared and distinct genes between cardiomyocytes isolated by Langendorff or TSAD. **(D)** qPCR validation of cardiomyocyte contraction-, fatty acid metabolism-, and ion channel-related genes. Data are mean ± SEM.

Tissue sectioning and mechanical agitation of the digestion solution is thought to induce stress that can be detrimental to cells. To determine whether such mechanical stress perturbed gene expression, we performed RNA-seq on isolated LV-, RV-, and LAA-CMs. Neither LV- nor RV-CMs exhibited any differential gene expression between these two methods (Fig 3A). In the LAA, the TSAD group displayed upregulation of the expression of only 5 genes, including three heatshock proteins (*HSP70*.*2*, *HSPA1L*, *HSPH1*), *CD40* (tumor necrosis factor receptor superfamily member 5), and *ERF* (ETS2 repressor factor) (Fig 3A), suggesting TSAD induced minimal levels of stress compared to Langendorff perfusion.

To analyze gene expression in greater depth, we extracted the top 500 genes in each sample, and overlapped them pairwise (Fig 3B). The number of overlapping genes were 466, 466, and 462, for LV, RV and LAA, respectively, indicating high similarities in the genes expression profiles of cardiomyocytes isolated with Langendorff or TSAD (Fig 3B).

Then, we performed functional enrichments (Kyoto Encyclopedia of Genes and Genomes (KEGG) and Gene Ontology (GO)) of shared and distinct genes, respectively, for each compartment (Fig 3C, S4–S12 Data, S1 Fig). According to GO analysis, the top 5 shared pathways between Langendorff and TSAD groups in LV, RV, and LAA, were identical, which included translation, generation of precursor metabolites and energy, peptide metabolic process, cellular respiration, and oxidation-reduction process, suggesting sufficient preservation of key aspects of cardiomyocyte function via either method (S1 Fig). KEGG analysis revealed similar results, oxidative phosphorylation, cardiac muscle contraction, citrate cycle (TCA cycle), and adrenergic signaling in cardiomyocytes among the most highly enriched terms (Fig 3C). These data show that the key function-related genes are similarly expressed regardless of isolation method. Then, we inspected genes specific to the Langendorff method. Surprisingly, based on KEGG analysis, CMs from each of the compartments showed functional enrichment in apoptosis (e.g., *GADD45G*, *DDIT3*, *ACTB*) (Fig 3C). The LV even exhibited involvement in ferroptosis (Fig 3C). While Langendorff-isolated LV- and LAA-CMs did not show enrichment in any GO biological process, genes in the RV displayed marked enrichment of apoptosis (Fig 3C, S1 Fig). These findings indicated that Langendorff perfusion may facilitate apoptosis. Interestingly, genes specific to TSAD demonstrated strong ties to Src-like adaptor proteins (e.g., *SLA-1*, *SLA-2*, *SLA-3*, *SLA-5*, *HSPA6*, *SLA-DRA*) and processes related to antigen processing and presentation.

We further validated the expression of a set of cardiac contraction-, fatty acid metabolism-, and ion channel-related genes. In accordance with RNA-seq results, none of the examined genes displayed differences between these two methods, regardless of anatomical region (Fig 3D, S13 Data). Interestingly, *KCNH2*, the gene underlying the delayed rectifier current (*I*_kr_), was highly expressed in ventricular CMs, but was largely undetectable in LAA CMs, indicating electrophysiological differences between CMs from these different compartments.

### 3.4. Cardiomyocyte electrophysiology

**Fig 4:**
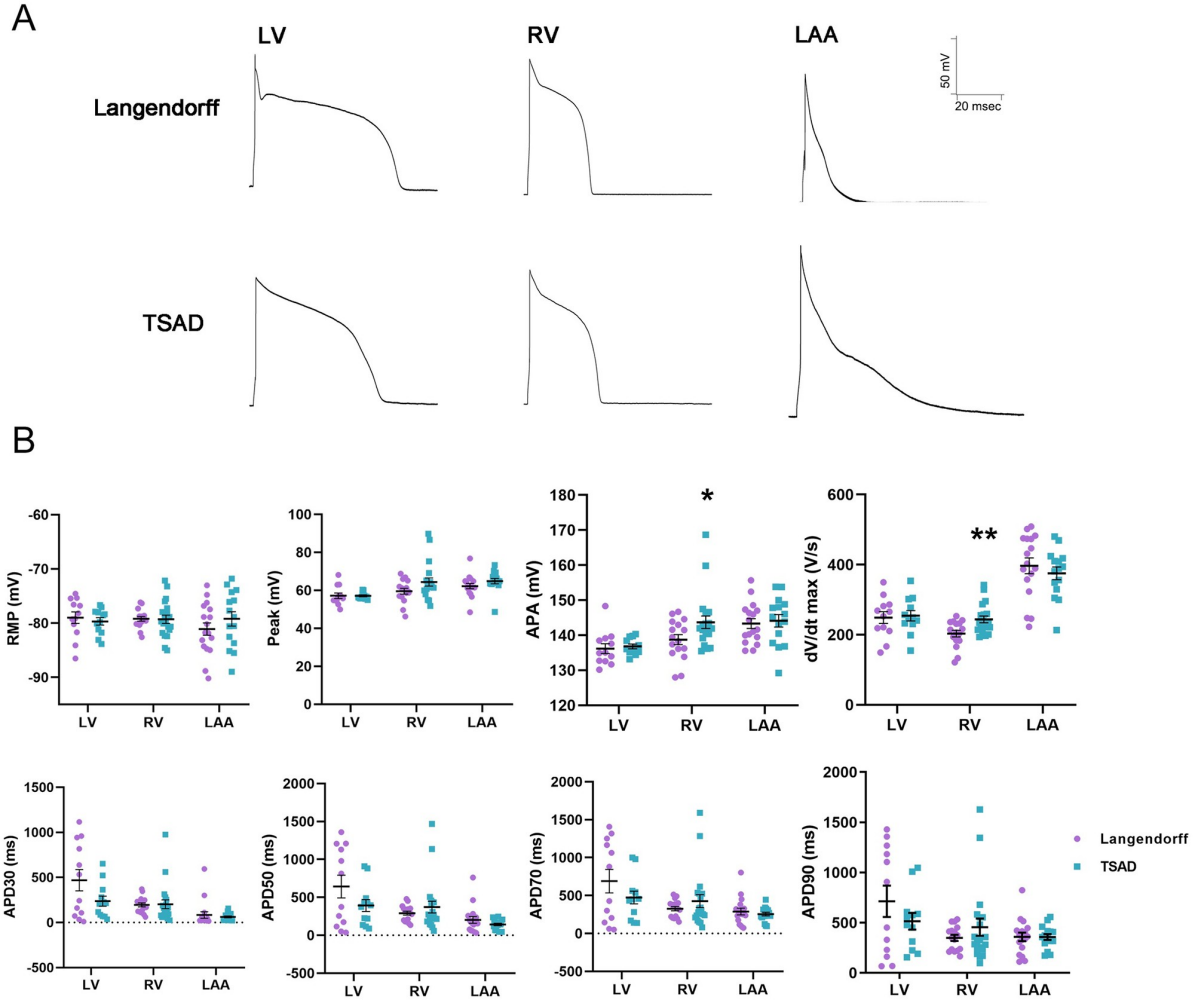
Action potential recordings of porcine cardiomyocytes. **(A)** Whole-cell patch clamping of freshly isolated porcine CMs. **(B)** Quantification of action potentials. Action potential parameters, including action potential amplitude (APA), peak, rate of membrane depolarization (dV/dtmax), resting membrane potential (RMP), and action potential durations at 30% (APD30), 50% (APD50), 70% (APD70) and 90% (APD90) repolarization, were quantified. Data are mean ± SEM. * *P* < 0.05, ** *P* < 0.01, two-way ANOVA, compared to Langendorff.

Finally, to assess cardiomyocyte function, we performed whole-cell patch clamping on freshly isolated cells (Fig 4A). Action potential tracings of ventricular CMs all displayed typical shapes with plateaus, while those of atrial CMs were narrower with diminished plateaus. Action potential parameters, including resting membrane potential (RMP), peak, action potential amplitude (APA), rate of membrane depolarization (dV/dtmax), and action potential durations at 30% (APD30), 50% (APD50), 70% (APD70) and 90% (APD90) repolarization, were similar between the two methods, regardless of the anatomical region (except for higher APA and dV/dtmax in TSAD-isolated RV-CMs), indicating TSAD isolation produced CMs with proper electrophysiology, suggesting functional integrity (Fig 4B, S14 Data).

## 4. Discussion/conclusions

Although Langendorff perfusion remains the to-go method of cardiomyocyte dissociation from the adult mammalian heart, particularly rodents, it is not applicable to the isolation of adult human cardiomyocytes, or those from larger mammals, such as the pig. Therefore, chunk digestion methods were applied to resected myocardial specimens. The performance of chunk digestion methods was variable among laboratories, and yielded cardiomyocytes of relatively low quality [8–13]. Our group previously reported on the use of tissue slicing-based chunk digestion method for the isolation adult human primary cardiomyocytes [2], which was later further optimized to yield a full set of methodology [3]. In this study, we sought to investigate how this method compares to Langendorff perfusion. Our data suggest that TSAD-isolated cardiomyocytes displayed qualities largely indistinguishable from to those of Langendorff perfusion, implying it as a valid surrogate when Langendorff perfusion is not feasible.

Our study is, to our knowledge, the first to compare a set of parameters of cardiomyocyte quality between TSAD and Langendorff from different cardiac compartments in large animals. Yue et al. compared the effects of chunk and perfusion methods on potassium currents in the isolation of canine atrial cardiomyocytes [14]. The authors observed similar transient outward currents in chunk- and perfusion-digested cardiomyocytes. However, the chunk method was found to significantly suppress *I*_kr_, the mechanism of which was unknown. They attributed this effect to the specific isolation method. In their protocol, the specimens were chopped into cube-shaped pieces approximately 1.5 mm^3^ in size, which might have limited enzyme perfusion, thus impairing cell quality. Our method generates 300 μm-thick tissue slices, which are further cut into tiny tissue fragments. This allows sufficient exposure of cardiomyocytes to the enzymatic solution, promoting cell viability and improving cell quality. We observed that *I*_kr_ was preserved by either method in ventricular CMs, but was barely detectable in LAA-CMs, indicating that ventricular and atrial CMs utilize different currents for repolarization [15–17].

The use of vibratome to cut myocardial biopsies for the isolation of cardiomyocytes dates back to 1995 [12], when slicing was performed by clamping the tissue between two pieces of polystyrene foam at room temperature without any bath solution. The resulting cell yield was very low, i.e., 1–5%, which could be a possible reason why later studies did not continue to pursue TSAD. The superior performance of our protocol could be due to the immersion of the myocardial specimen in ice-cold cardioplegic buffer during the entire slicing procedure [2]. A recent study reported the use of agarose for the embedding of myocardial specimen to facilitate slicing [18]. However, the reported cell yield of 200 cells per mg of tissue, was still significantly lower than our reported yield of 3.28 ×10^6^/gram of tissue [3]. This was probably due to the inability of the cutting solution to get into contact with the myocardium when the tissue was embedded in agarose.

In summary, our TSAD protocol for the isolation of adult human primary cardiomyocytes was applicable to adult porcine cardiomyocytes, and the resulting cell quality was on a par with Langendorff perfusion. This comprehensive evaluation of CM quality provides compelling evidence of the utility and reliability of TSAD in translational studies.

## Supporting information

S1 Fig Comparison of different cell viability assessment methods. A. Representative images of isolated cardiomyocytes. In the LIVE/DEAD staining method, calcein labels live cells green, while EthD-1 labels dead cells red. In the PI staining method, dead cells are labeled red. LV indicates cardiomyocytes isolated from left ventricular myocardium (*n* = 1 patient), LAA indicates cardiomyocytes isolated from the left atrial appendage (*n* = 2 patients). Scale bar = 200 μm. B. Quantification of cell viabilities by LIVE/DEAD staining, PI staining and by percentage of rod-shaped cells. Data are mean ± SEM. * *P* < 0.05, paired Student’s *t*-test. C. Tissue mass-normalized yields of cardiomyocytes isolated via Langendorff or TSAD. n = 3 minipigs for each group. Data are mean ± SEM.
(PDF)

S2 Fig Functional enrichment (GO) of shared and distinct genes between cardiomyocytes isolated by Langendorff or TSAD. (PDF)

S1 Data Supporting data for cardiomyocyte viability and morphology quantification. Spreadsheet “Percent viable cells”: Quantification of the percentage viable cardiomyocytes (related to Fig 1C); Spreadsheet “Percent rod-shaped cells”: Quantification of the percentage rod-shaped cardiomyocytes (related to Fig 1D); Spreadsheet “Cell Length”: Q Quantification of cell length (related to Fig 1E).
(XLSX)

S2 Data Comparison of different cell viability assessment methods. Spreadsheet “Percentage”: Quantification of cell viabilities by LIVE/DEAD staining, PI staining and by percentage of rod-shaped cells (related to S1B Fig); Spreadsheet “Cell yield (×10^4) g tissue”: Quantification of tissue mass-normalized yields of cardiomyocytes isolated via Langendorff or TSAD.
(XLSX)

S3 Data Sarcomere and mitochondrial measurements. Spreadsheet “Sarcomere Length”: Quantification of the Sarcomere Length of cardiomyocytes (related to Fig 2B); Spreadsheet “Mitochondrial size”: Quantification of the Mitochondrial size of cardiomyocytes (related to Fig 2D).
(XLSX)

S4 Data Functional enrichment of shared top 500 genes between LV-CMs isolated via Langendorff or TSAD. Spreadsheet “kegg”: KEGG enrichment of shared top 500 genes between LV-CMs isolated via Langendorff or TSAD (related to Fig 3C). Spreadsheet “GO_BP”: GO enrichment of shared top 500 genes between LV-CMs isolated via Langendorff or TSAD (related to S1 Fig).
(XLSX)

S5 Data Functional enrichment of Langendorff-specific genes within the top 500 LV-CM genes. Spreadsheet “kegg”: KEGG enrichment of distinct top 500 genes LV-CMs isolated via Langendorff (related to Fig 3C).
(XLSX)

S6 Data Functional enrichment of TSAD-specific genes within the top 500 LV-CM genes. Spreadsheet “kegg”: KEGG enrichment of distinct top 500 genes LV-CMs isolated via TSAD (related to Fig 3C). Spreadsheet “GO_BP”: GO enrichment of distinct top 500 genes LV-CMs isolated via TSAD (related to S1 Fig).
(XLSX)

S7 Data Functional enrichment of shared top 500 genes between RV-CMs isolated via Langendorff or TSAD. Spreadsheet “kegg”: KEGG enrichment of shared top 500 genes between RV-CMs isolated via Langendorff or TSAD (related to Fig 3C). Spreadsheet “GO_BP”: GO enrichment of shared top 500 genes between RV-CMs isolated via Langendorff or TSAD (related to S1 Fig).
(XLSX)

S8 Data Functional enrichment of Langendorff-specific genes within the top 500 RV-CM genes. Spreadsheet “kegg”: KEGG enrichment of distinct top 500 genes RV-CMs isolated via Langendorff (related to Fig 3C). Spreadsheet “GO_BP”: GO enrichment of distinct top 500 genes RV-CMs isolated via Langendorff (related to S1 Fig).
(XLSX)

S9 Data Functional enrichment of TSAD-specific genes within the top 500 RV-CM genes. Spreadsheet “kegg”: KEGG enrichment of distinct top 500 genes RV-CMs isolated via TSAD (related to Fig 3C).
(XLSX)

S10 Data Functional enrichment of shared top 500 genes between LAA-CMs isolated via Langendorff or TSAD. Spreadsheet “kegg”: KEGG enrichment of shared top 500 genes between LAA-CMs isolated via Langendorff or TSAD (related to Fig 3C). Spreadsheet “GO_BP”: GO enrichment of shared top 500 genes between LAA-CMs isolated via Langendorff or TSAD (related to S1 Fig).
(XLSX)

S11 Data Functional enrichment of Langendorff-specific genes within the top 500 LAA-CM genes. Spreadsheet “kegg”: KEGG enrichment of distinct top 500 genes LAA-CMs isolated via Langendorff (related to Fig 3C).
(XLSX)

S12 Data Functional enrichment of TSAD-specific genes within the top 500 LAA-CM genes. Spreadsheet “kegg”: KEGG enrichment of distinct top 500 genes LAA-CMs isolated via TSAD (related to Fig 3C). Spreadsheet “GO_BP”: GO enrichment of distinct top 500 genes LAA-CMs isolated via TSAD (related to S1 Fig).
(XLSX)

S13 Data qPCR validation of RNA-seq data. Spreadsheet “Relative Quantitation-LV”: Quantification of relative mRNA expression levels of indicated genes in the LV (related to Fig 3D); Spreadsheet “Relative Quantitation-RV”: Quantification of relative mRNA expression levels of indicated genes in the RV (related to Fig 3D); Spreadsheet “Relative Quantitation-LAA”: Quantification of relative mRNA expression levels of indicated genes in the LAA (related to Fig 3D).
(XLSX)

S14 Data Action potential recordings of porcine cardiomyocytes. Spreadsheet “APD30”: Quantification of r action potential durations at 30% (APD30) (related to Fig 4B); Spreadsheet “APD50”: Quantification of r action potential durations at 50% (APD50) (related to Fig 4B); Spreadsheet “APD70”: Quantification of r action potential durations at 70% (APD70) (related to Fig 4B); Spreadsheet “APD90”: Quantification of r action potential durations at 90% (APD90) (related to Fig 4B); Spreadsheet “dVdt max”: Quantification of peak, rate of membrane depolarization (dV/dtmax) (related to Fig 4B); Spreadsheet “Peak”: Quantification of statistics of peak (related to Fig 4B); Spreadsheet “RMP”: Quantification of resting membrane potential (related to Fig 4B); Spreadsheet “APA”: Quantification of action potential amplitude (related to Fig 4B).
(XLSX)

S1 File Supplemental methods include human samples, S1 Table. patient information, propidium iodide staining, and calculation of the percentage of rod-shaped cells.
(PDF)
