# Supplementary material for: Isolation of porcine adult cardiomyocytes: Comparison between Langendorff perfusion and tissue slicing-assisted enzyme digestion
Source: PLoS One. 2023 May 26;18(5):e0285169. doi: 10.1371/journal.pone.0285169 (PMC10218724; doi:10.1371/journal.pone.0285169)
Supplement: S1 Fig — A. Representative images of isolated cardiomyocytes. In the LIVE/DEAD staining method, calcein labels live cells green, while EthD-1 labels dead cells red. In the PI staining method, dead cells are labeled red. LV indicates cardiomyocytes isolated from left ventricular myocardium (n = 1 patient), LAA indicates cardiomyocytes isolated from the left atrial appendage (n = 2 patients). Scale bar = 200 μm. B. Quantification of cell viabilities by LIVE/DEAD staining, PI staining and by percentage of rod-shaped cells. Data are mean ± SEM. * P < 0.05, paired Student’s t-test. C. Tissue mass-normalized yields of cardiomyocytes isolated via Langendorff or TSAD. n = 3 minipigs for each group. Data are mean ± SEM. (PDF) [file pone.0285169.s001.pdf]

S1 Fig

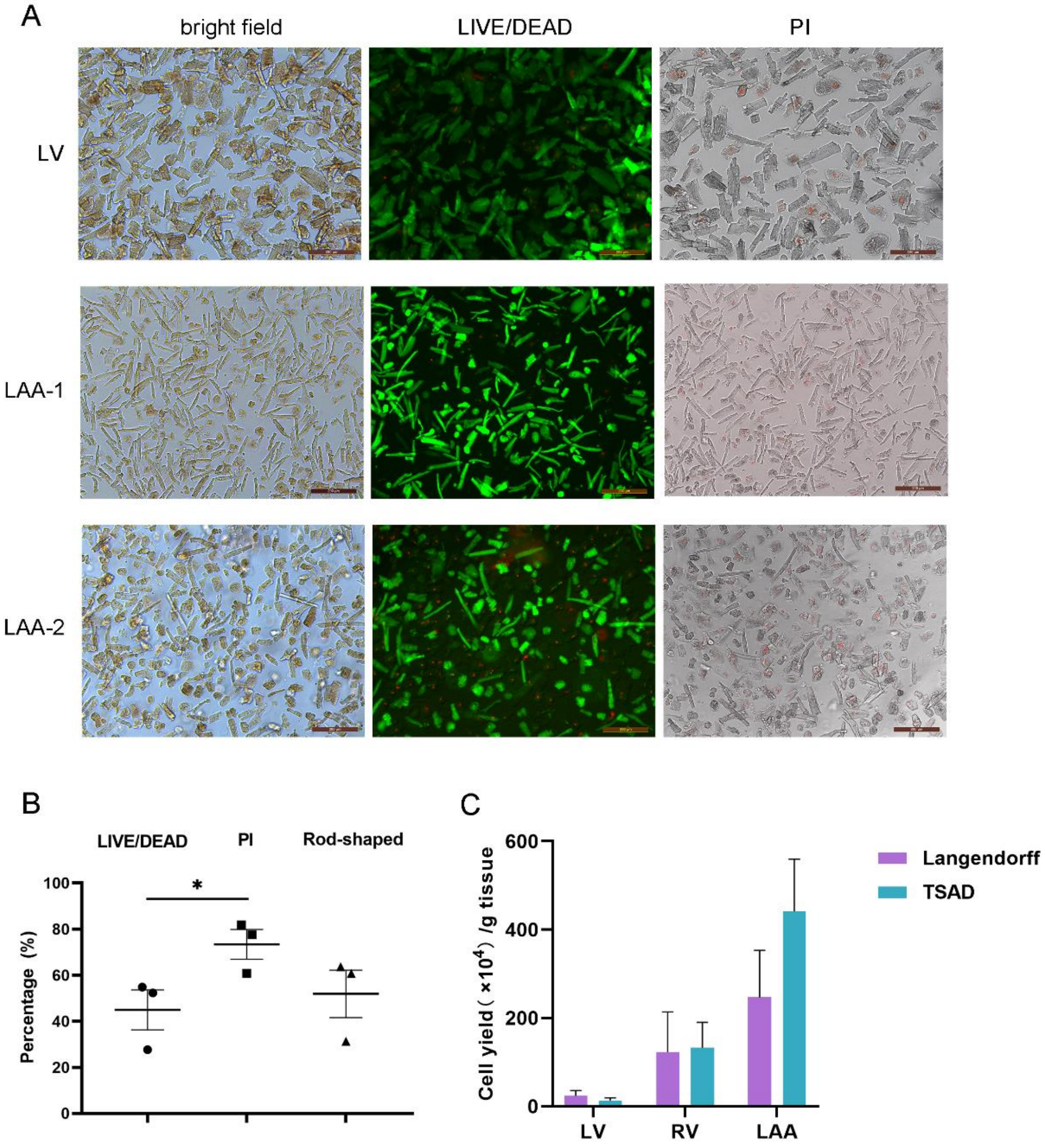

S1 Fig. Comparison of different cell viability assessment methods. **A**. Representative images of isolated cardiomyocytes. In the LIVE/DEAD staining method, calcein labels live cells green, while EthD-1 labels dead cells red. In the PI staining method, dead cells are labeled red. LV indicates cardiomyocytes isolated from left ventricular myocardium (n = 1 patient), LAA indicates cardiomyocytes isolated from the left atrial appendage (n = 2 patients). Scale bar = 200 μm. **B**. Quantification of cell viabilities by LIVE/DEAD staining, PI staining and by percentage of rod-shaped cells. Data are mean ± SEM. * $P < 0.05$, paired Student's t-test. **C**. Tissue mass-normalized yields of cardiomyocytes isolated via Langendorff or TSAD. n =3 minipigs for each group. Data are mean ± SEM.
