# Supplementary material for: Isolation of porcine adult cardiomyocytes: Comparison between Langendorff perfusion and tissue slicing-assisted enzyme digestion
Source: PLoS One. 2023 May 26;18(5):e0285169. doi: 10.1371/journal.pone.0285169 (PMC10218724; doi:10.1371/journal.pone.0285169)
Supplement: S2 Fig — (PDF) [file pone.0285169.s002.pdf]

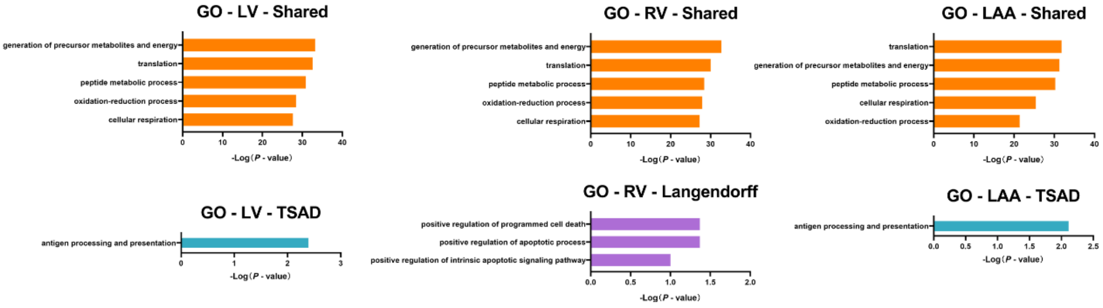

S2 Fig. Functional enrichment (GO) of shared and distinct genes between cardiomyocytes isolated by Langendorff or TSAD.
