# Supplementary material for: Isolation of porcine adult cardiomyocytes: Comparison between Langendorff perfusion and tissue slicing-assisted enzyme digestion
Source: PLoS One. 2023 May 26;18(5):e0285169. doi: 10.1371/journal.pone.0285169 (PMC10218724; doi:10.1371/journal.pone.0285169)
Supplement: S1 File — patient information, propidium iodide staining, and calculation of the percentage of rod-shaped cells. (PDF) [file pone.0285169.s017.pdf]

Supplementary Materials

Isolation of porcine adult cardiomyocytes: Comparison between Langendorff perfusion and tissue slicing-assisted enzyme digestion

Xun Shi, Xiaoli Tang, Fang Yao , Le Wang, Mingzhi Zhang, Xin Wang, Guangxin Yue, Li Wang, Shengshou Hu*, Bingying Zhou*

This file includes the following:

Supplemental Methods

Supplemental Figures 1-2

Supplemental Methods

Human samples

hPCMs used in this study were isolated from the left ventricle of patients undergoing Morrow surgery, or the left atrial appendages of patients undergoing coronary artery bypass graft. Written informed consent was obtained from all patients. The study was approved by the Ethics Committee of Fuwai Hospital, Chinese Academy of Medical Sciences and Peking Union Medical University, and conducted according to the Declaration of Helsinki.

Supplemental Table 1. Patient Information.

patient ID	age	sex	primary diagnosis	surgery	tissue source
001-001	52	M	Hypertrophic obstructive cardiomyopathy	Morrow	LV
001-002	58	M	Coronary atherosclerotic heart disease	CABG	LAA
001-003	62	M	Coronary atherosclerotic heart disease	CABG	LAA

Propidium iodide staining

Propidium iodide (Thermo P3566, 1mg/ml) was purchased and used following the manufacturer's instructions. In brief, the stock solution was diluted 500-fold, and 2 µl of the working solution was added to 1 ml of cell suspension (3×10^4 cells). Following 15 min of incubation at room temperature protected from light, the cell suspension was gently centrifuged at $100 \times g$ for 1 min, and the cell pellet was washed once with Tyrode's solution. The final cell pellet was resuspended in 200 µl of Tyrode's solution, plated into a well of a 48-well plate, and imaged using a fluorescent microscope (excitation/emission: 493/636nm). Cell viability was calculated as the fraction of unstained cells within the total cell population.

Calculation of the percentage of rod-shaped cells

Bright-field cell images were counted manually using Image J. The percentage of rod-shaped cells was calculated as the fraction of cells maintaining the rod shape within the total cell population.
labeled red. LV indicates cardiomyocytes isolated from left ventricular myocardium (*n* = 1 patient), LAA indicates cardiomyocytes isolated from the left atrial appendage (*n* = 2 patients). Scale bar = 200 µm. **B**. Quantification of cell viabilities by LIVE/DEAD staining, PI staining and by percentage of rod-shaped cells. Data are mean ± SEM. * $P < 0.05$, paired Student's *t*-test. **C**. Tissue mass-normalized yields of cardiomyocytes isolated via Langendorff or TSAD. n =3 minipigs for each group. Data are mean ± SEM.
